# Ethanol sclerotherapy for endometriomas: a fertility-preserving alternative

**DOI:** 10.3389/frph.2025.1716957

**Published:** 2025-12-04

**Authors:** Johnny S. Younis

**Affiliations:** 1 Department of Obstetrics and Gynecology, Tzafon Medical Center, Reproductive Medicine, Poriya, Israel; 2 Azrieili Faculty of Medicine in Galilee, Bar-Ilan University, Safed, Israel

**Keywords:** fertility preservation, endometrioma, ethanol sclerotherapy, ovarian reserve, endometriosiis

## Abstract

Endometriomas are a common manifestation of endometriosis in women of reproductive age and pose a clinical challenge due to their association with pain, infertility, and compromised ovarian reserve. Surgical removal through cystectomy remains the standard intervention, but compelling evidence demonstrates its deleterious impact on ovarian reserve and potential acceleration of ovarian aging. These concerns have prompted an investigation of less invasive alternatives. Among these, ethanol sclerotherapy has emerged as a promising, minimally invasive, often ultrasound-guided procedure offering cyst resolution with minimal trauma to ovarian tissue. This mini-review synthesizes current evidence on ethanol sclerotherapy for the management of endometriomas, with an emphasis on clinical outcomes and implications for fertility preservation. Evidence indicates that ethanol sclerotherapy is highly effective technically, with low rates of major complications. Pain relief is achievable, recurrence rates can be reduced with longer ethanol exposure, and ovarian reserve is preserved compared with cystectomy. Assisted reproduction outcomes suggest comparable pregnancy rates, with some data supporting a higher oocyte yield following sclerotherapy. Nevertheless, the quality of evidence is limited, predominantly derived from observational studies, and results vary regarding long-term efficacy and reproductive outcomes. Ethanol sclerotherapy is best considered a minimally invasive, fertility-sparing option for women seeking to avoid surgery or preserve reproductive potential. Future randomized controlled trials should clarify its role relative to cystectomy and expectant management, establish optimal procedural parameters, and assess long-term outcomes, including ovarian reserve, live birth rates, and cost-effectiveness.

## 1 Introduction

Ovarian endometriomas are a frequent manifestation of endometriosis in reproductive-age women, affecting up to 55% of those with the condition (1). They pose a significant clinical challenge due to their association with pain, infertility, and decreased ovarian reserve (2–4). Advances in imaging, particularly ultrasound, now enable a reliable diagnosis in most cases (5); however, management remains debated, especially in women who wish to preserve fertility (6, 7). Although cystectomy continues to be regarded as the standard of care, accumulating evidence demonstrates its detrimental effect on ovarian reserve, with the potential to accelerate ovarian aging (8–10).

These concerns have shifted attention toward fertility-preserving strategies. Minimally invasive options, such as ablative techniques or aspiration combined with sclerotherapy, aim to manage endometriomas while sparing healthy ovarian tissue. Among the sclerosing agents investigated, ethanol has been most widely adopted due to its potent cytotoxic, dehydrating, and thrombogenic effects on endometriotic cyst walls, as well as low affordability (11). First described more than thirty years ago, ethanol sclerotherapy for endometrioma management today is commonly performed under ultrasound guidance, offering a minimally invasive approach that may reduce damage to ovarian reserve compared with conventional cystectomy (12–14).

Ethanol sclerotherapy has been applied both as primary management and as treatment for recurrent endometriomas. Its feasibility in outpatient or day-surgery settings, combined with relatively low complication rates, makes it particularly attractive. Yet, uncertainties remain regarding optimal technique, durability of results, and long-term reproductive outcomes (15).

This mini-review summarizes clinical evidence on ethanol sclerotherapy, focusing on safety, efficacy, impact on ovarian reserve, and fertility outcomes. In doing so, it highlights the potential of this approach as a fertility-preserving alternative to cystectomy and identifies key gaps requiring further investigation.

## 2 Mechanism and procedure

Ethanol sclerotherapy involves aspiration of endometrioma contents followed by flushing with normal saline solution, and instillation of ethanol into the cyst cavity under ultrasound guidance, most commonly transvaginally. Ethanol acts by denaturing proteins, dehydrating epithelial cells, and inducing coagulative necrosis of the cyst wall. Vascular thrombosis within the cyst lining further contributes to ablation of the endometriotic tissue (11). In line with interventional radiology experience, the total ethanol dose is commonly limited to approximately 1 mL/kg, as doses in this range have been associated with systemic blood alcohol concentrations up to about 0.07% and a potential risk of intoxication (11).

For sclerotherapy, ethanol is typically used in high-concentration, parenteral-grade formulations. Commercial products, such as Dehydrated Alcohol Injection, USP and Dehydrated Alcohol for Injection BP, are sterile, non-denatured ethanol solutions containing ≥98%–100% ethanol by volume, supplied as preservative-free single-dose ampules or vials (usually 1–5 mL) intended for injection (16). In ovarian endometrioma sclerotherapy, published protocols most commonly employ 95%–96% or 99% ethanol, with the instilled volume defined as a fraction of the aspirated cyst volume, sometimes with an absolute upper cap, rather than on a per-kilogram body-weight basis. Only sterile, non-denatured preparations specifically formulated for parenteral use should be used; laboratory-grade or industrial “absolute ethanol” is unsuitable. The use of denatured alcohol is contraindicated because it contains toxic additives such as methanol and other denaturants that are unsafe for parenteral administration. When lower concentrations are desired, some protocols have described ethanol diluted to 50%–70% under aseptic conditions by hospital pharmacy. These considerations outline the characteristics of parenteral ethanol preparations used in clinical practice for this off-label procedure.

The technical aspects of endometrioma ethanol sclerotherapy vary widely across published studies. Ethanol concentrations range from 20% to 100%, though most protocols use 95%–100% (17–20). The instilled volume typically corresponds to 20%–100% of the aspirated cyst volume, not exceeding 60–100 mL to minimize ethanol spillage (17, 19, 20). The duration of ethanol contact varies: some protocols recommend immediate aspiration after irrigation (“wash-out”), while others suggest retention for 5–20 min. Increasing evidence suggests that retention for longer than 10 min results in lower recurrence compared with shorter exposure or wash-out techniques (17, 18).

Procedures are usually performed transvaginally under ultrasound guidance. However, the transabdominal approach (using abdominal ultrasound or laparoscopy) is employed in select cases depending on the endometrioma's location, accessibility, and specific case features. Notably, the laparoscopic approach offers the advantage of direct visualization, enabling immediate cleaning and irrigation of the pelvis in the event of ethanol spillage, thereby minimizing inflammation and adhesions (21).

The transvaginal procedure is typically carried out after cleansing the vagina under sterile conditions. The endometrioma is usually punctured, its content aspirated, and flushed using an echogenic needle ranging from 17 to 20 gauge and 20–25 cm long (18–20, 22). Local analgesia, sedation, or general anesthesia is usually required; some centers have recently reported the feasibility of outpatient settings without anesthesia (23–25).

Overall, ethanol sclerotherapy, especially the transvaginal approach, is a technically straightforward, broadly accessible, and repeatable procedure. However, heterogeneity in technique underscores the need for standardized protocols to optimize safety and effectiveness.

## 3 Clinical outcomes

### 3.1 Safety and adverse events

Available evidence consistently indicates that ethanol sclerotherapy is a safe treatment. Minor adverse events (pelvic pain, fever, mild bleeding, ethanol leakage) occur in about 10% of cases, while major complications (abscess formation, ethanol intoxication) are reported in fewer than 2% (18, 22, 26). To reduce major complications, specifically intoxication, intra-procedural ethanol loss should be meticulously evaluated, and test blood alcohol levels if leakage is suspected. Endometriomas larger than 80 mm should be managed in specialized centers (27). While prophylactic antibiotics are advised in these cases, they do not appear to prevent infectious complications (27). Overall, compared to cystectomy, complication rates are at least comparable, with the advantage of avoiding surgical risks such as inadvertent ovarian tissue removal or adhesion formation.

In the 8-year single-center experience by Miquel et al. involving 126 women, ethanol sclerotherapy was associated with an overall complication rate of 9.5%, including mild pelvic pain (6%) and transient fever (2%), with one case of ethanol intoxication reported leading to coma (27). In this case, the maximum blood alcohol level was 2.38 g/L and the ethanol loss was 30 mL. Kim et al. pooled 21 studies that employed ethanol and found a major complication rate of 1.7%, primarily abscess formation (18). Frankowska et al. observed only isolated transient pain events in their review of 16 studies, confirming a high safety profile (22). In contrast, García-García et al. reported ethanol leakage in 3.1% of procedures, yet without systemic toxicity (26). Collectively, these data confirm that ethanol sclerotherapy is a low-risk intervention when performed with ultrasound guidance and careful monitoring of ethanol loss.

### 3.2 Technical efficacy

Across published systematic reviews, ethanol sclerotherapy demonstrates high technical success rates, typically ranging from 95% to 98% (18, 19). The high success rate reflects the ability to aspirate, perform adequate saline flushing, and successfully instill ethanol without procedural failure. Success is largely influenced by factors such as cyst accessibility, operator skill, and the duration of ethanol exposure. However, in a recent comprehensive single-center retrospective cohort study involving 126 women and 131 procedures, the reported failure rate was approximately 10% (27). The main reasons for failure were saline solution leakage, indicating endometrioma rupture during flushing, interpositions of the digestive tract, and thick endometrioma content that could not be adequately aspirated.

### 3.3 Pain relief

Overall, sclerotherapy has been shown to improve endometriosis-associated pain; however, the success rate has not been consistently reported across systematic reviews (17, 18, 20, 22). In recent single-center retrospective low-scale studies, significant improvement in dysmenorrhea and pelvic pain was reported within 6–12 months following the procedure (28, 29). In the meta-analysis by Kim et al, the pooled pain-relief rate was 85.9% (95% CI 73.9%–92.9%, *I*^2^ = 48%), similar to cystectomy but achieved through a minimally invasive approach (18). Long-term follow-up data remain limited beyond one year. While cystectomy also provides effective pain relief, ethanol sclerotherapy offers a less invasive alternative with potentially minimal tissue damage (22).

### 3.4 Recurrence

Recurrence remains the main limitation of conservative approaches. Rates after ethanol sclerotherapy vary widely, from 10% to 60% depending on follow-up duration and technique (17, 22). In the systematic review by Kim et al., a pooled estimate of 13.8% (9.4%–19.9%; *I*^2^ = 75%) was found (18). Retention of ethanol for more than 10 min significantly reduces recurrence compared to shorter exposure or irrigation techniques (17, 18). While recurrence rates may be slightly higher than after cystectomy, repeated sclerotherapy is feasible and does not seem to entail cumulative surgical trauma (30).

### 3.5 Ovarian reserve

Preservation of ovarian reserve is arguably the primary benefit of ethanol sclerotherapy over surgical excision. Whereas cystectomy consistently leads to a substantial reduction in serum AMH levels, estimated at 40%–60% after one year, with greater losses after bilateral procedures (8, 31), two systematic reviews indicate that ethanol sclerotherapy does not significantly alter AMH levels (18, 22). A recent systematic review further confirmed that sclerotherapy maintains ovarian reserve more effectively than endometriotic cystectomy, as reflected by a smaller decline in AMH (32).

In a recent single-center retrospective comparison study (*n* = 70), laparoscopic endometriotic cystectomy caused a significant reduction in AMH levels after 12 months (2.48 ± 1.34 vs. 1.62 ± 1.22; *P* < 0.001), whereas ethanol sclerotherapy did not impact serum AMH levels (2.12 ± 1.05 vs. 2.09 ± 1.01; *P* = 0.120) (33). In the systematic review by Kim et al., no significant overall change in AMH was observed, with a mean difference of −0.01 ng/mL (−0.04–0.03, *P* = 0.95, *I*^2^ = 0%) (18). Lavadia et al. demonstrated a significantly greater decline in AMH after endometriotic cystectomy compared to sclerotherapy, with a mean difference of 1.69 ng/mL (95% CI 0.58–2.80, *P* = 0.003, *I*^2^ = 94%) (32). These consistent findings seem to highlight the ovarian-reserve–sparing benefit of ethanol exposure compared to endometriotic cystectomy.

Several reviews have also reported improved AFC following sclerotherapy, possibly reflecting decompression of the ovary once the cyst decreases considerably in size (17, 22, 34, 35). However, this should be interpreted with caution, as AFC is a less sensitive measure of ovarian reserve than AMH in cases with endometrioma before surgical management (36).

A theoretical concern is that ethanol might diffuse through the cyst wall into the adjacent ovarian cortex, potentially exposing primordial follicles. Experimental animal studies using rat models, which investigate the injection of ethanol into simple ovarian cysts, reveal stromal fibrosis and follicular loss (37, 38). However, there is no experimental model that measures ethanol diffusion from an endometrioma cavity into ovarian tissue, and the depth of ethanol penetration remains unknown. Nevertheless, prolonged ethanol exposure has been linked to greater tissue injury in animal models, and clinical series highlight that minimizing dwell time, ensuring complete re-aspiration, avoiding leakage, and carefully monitoring ethanol loss appear to mitigate the risk of diffusion-related damage to ovarian reserve.

Collectively, although current evidence suggests that ethanol sclerotherapy is less damaging to ovarian reserve than endometriotic excision, long-term data are still lacking. Furthermore, there is a lack of robust data on how ethanol concentration and sclerosing duration impact ovarian reserve measures.

### 3.6 Reproductive outcomes

Pregnancy outcomes following ethanol sclerotherapy appear promising, although evidence is heterogeneous and based mainly on small observational studies. Reported overall pregnancy rates range from 30% to 40%, with no definitive difference between spontaneous and assisted conceptions (17–19, 22, 39). In cases of assisted reproduction, sclerotherapy yields similar numbers of retrieved oocytes and clinical pregnancy rates compared to no intervention (17, 22, 39).

Notably, Cohen et al. observed a higher average oocyte yield with sclerotherapy compared to endometriotic cystectomy with a standardized mean difference of 2.71 (95% CI 0.98–4.44, *P* = 0.03, *I*^2^ = 70%) (17). Similar findings were reported in a recent meta-analysis by Lavadia, which showed higher clinical pregnancy rates after ethanol treatment (OR 1.80, 95% CI 1.24–2.60, *P* = 0.01, *I*^2^ = 50%) with comparable live birth outcomes (32). These results suggest that sclerotherapy preserves ovarian responsiveness without impairing implantation potential. However, evidence on live birth rates remains limited, primarily derived from small, observational studies. Conversely, He et al. found a non-significant difference in overall pregnancy rates (OR, 1.67; 95% CI 0.74–3.75; *P* = 0.22, *I^2^* = 34%), underscoring the need for standardized protocols for ethanol sclerotherapy (19).

## 4 Comparison with endometriotic cystectomy

Cystectomy has long been considered the standard treatment for endometriomas, as it allows for the tentative removal of the cyst and provides histological confirmation. However, it carries risks such as loss of ovarian reserve, surgical complications, and recurrence rates that can approach 50% after five years (40, 41). Table 1 summarizes the main points of comparison between the two approaches. Ethanol sclerotherapy offers advantages in several areas, including lower complication rates that are comparable to surgery. Its technical success is high, although recurrence tends to be greater unless ethanol is retained (17, 18, 20). Most notably, ethanol sclerotherapy generally preserves ovarian reserve, unlike cystectomy, which is associated with a decline (22, 31). Additionally, fertility outcomes appear similar, with some evidence of higher oocyte yields and improved clinical pregnancy rates during IVF cycles (32, 40). Furthermore, small-scale studies suggest that sclerotherapy may be more cost-effective, as it could reduce hospitalization and overall expenses (23, 24). Nonetheless, cystectomy remains necessary in cases of suspected malignancy, large symptomatic cysts, or when histological confirmation is required. Therefore, ethanol sclerotherapy should not be viewed as a universal replacement for endometriotic cystectomy but rather as a feasible and viable fertility-preserving option for women of reproductive age, especially those with infertility or planning a future pregnancy.

**Table 1:** Comparison of ethanol sclerotherapy versus endometriotic cystectomy^a^.

Aspect	Ethanol sclerotherapy	Endometriotic cystectomy
Safety	Minor complication 10% (pain, fever, ethanol leakage)	Surgical risks: bleeding, adhesions, ovarian tissue removal; complications depend on the surgeon's expertise
	Major complications <2% (ethanol intoxication, abscess formation)	
Technical efficacy	90%–98%	High (definitive removal, histology available)
Recurrence	10%–60%, lower with ethanol retention >10 min	20%–50% at 5 years; high repeat surgery rate
Ovarian reserve	Largely preserved (stable AMH, sometimes AFC increase)	40%–60% AMH decline at one year
Fertility outcomes	Comparable pregnancy rates; higher oocyte yield in IVF	Comparable pregnancy rate, lower oocyte yield in IVF
Recovery	Minimally invasive, rapid recovery, outpatient feasible	Requires operating room, hospitalization
Cost-effectiveness	Lower costs	Higher costs (surgery, hospitalization)
Limitations	No histology, high recurrence if short exposure, challenging if endometrioma >8 cm, contraindicated if suspicion of malignancy, and cyst rupture during the procedure	Invasive, loss of ovarian reserve, high recurrence over time

a Most available data on ethanol sclerotherapy are limited to short-term follow-up, especially on ovarian reserve and recurrence rate, whereas evidence on endometriotic cystectomy also includes long-term outcomes.

## 5 Future directions and research gaps

Despite encouraging outcomes, ethanol sclerotherapy remains underutilized in the management of ovarian endometriomas, largely due to the paucity of high-quality evidence and the heterogeneity of existing studies. Several important questions require clarification and study before this technique can be more widely adopted. Table 2 outlines key areas for future research in this field.

**Table 2:** Key areas for future research in ethanol sclerotherapy for ovarian endometriomas.

Research area	Current gap	Suggested focus
Primary versus Secondary Management	Data often mix primary (un-operated) and secondary (recurrent) endometrioma, leading to bias	Trials stratifying outcomes for primary versus recurrent endometrioma
Technique Standardization	Wide variation in ethanol concentration, volume, and sclerosing time	RCTs to define optimal ethanol concentration (e.g., 95%–100%), instillation volume, and exposure time (e.g., >10 min)
Patient selection	Influence of cyst size, laterality, and coexisting superficial/deep endometriosis not systemically addressed	Subgroup analysis and multi-center collaboration to stratify results by cyst characteristics and coexisting disease
Ovarian Reserve	Most studies are short-term; AMH change after sclerotherapy uncertain in long-term	Long-term monitoring of AMH and AFC post-procedure, impact of ethanol dose and exposure on reserve
Reproductive Outcome	Data mostly limited to pregnancy/clinical pregnancy; live birth rarely reported	Prospective studies with live birth as the primary endpoint, both spontaneous and ART-related
Long-term Efficacy	Limited follow-up, recurrence rates vary widely (10%–60%)	Standardized follow-up protocols to assess durability of treatment and feasibility of repeat sclerotherapy
Safety Profile	Adverse events inconsistently reported; systemic ethanol absorption and safety thresholds unclear	Standardized reporting of complications and pharmacokinetic studies to establish safe ethanol volume and concentration
Cost-Effectiveness	Minimally invasive suggests reduced costs, but no formal analysis available	Health-economic evaluation comparing sclerotherapy, cystectomy, and expectant management

A central issue is that available data combine cases of primary (unoperated endometrioma) and secondary (endometrioma recurrence) ethanol sclerotherapy, which causes bias, complicates the proper assessment of the procedure's safety and efficacy, and obscures its optimal position in the therapeutic sequence. Technique optimization also remains unresolved, with variation in ethanol concentration, instilled volume, and exposure duration across studies; identifying standardized parameters is critical for ensuring reproducible outcomes. Patient selection is another key area of uncertainty, as the influence of cyst size, laterality, and the presence of coexisting deep or superficial endometriosis on efficacy and safety has not been systematically evaluated. Furthermore, while short- and medium-term outcomes are encouraging, evidence on long-term efficacy one year and beyond is scarce, particularly with respect to recurrence and sustained reproductive performance. Similarly, attention should be focused on the impact of ethanol sclerotherapy on ovarian reserve measures over time, employing serum AMH, the most sensitive biomarker in this setting (36).

Furthermore, fertility endpoints reported to date are mostly limited to pregnancy and clinical pregnancy rates, whereas live birth, the most meaningful outcome for affected women, remains insufficiently studied. Finally, the outpatient feasibility of sclerotherapy, which may avoid general anesthesia and reduce hospitalization, raises the possibility of substantial health economic advantages; however, formal cost-effectiveness analyses are lacking. Addressing these gaps through well-designed randomized controlled trials and prospective cohort studies is essential to establish the precise role of ethanol sclerotherapy in endometrioma management and as a fertility-preserving alternative, and to position it appropriately alongside cystectomy and expectant management within individualized, multidisciplinary fertility-oriented care.

## 6 Conclusion

Ethanol sclerotherapy represents a safe, technically effective, and fertility-preserving alternative for managing ovarian endometriomas in reproductive-age women. It provides effective symptom relief, reduces recurrence when optimized, and crucially avoids the significant loss of ovarian reserve associated with cystectomy. Reproductive outcomes appear at least comparable, with a potential advantage in ovarian response during IVF.

However, the strength of evidence remains low, and uncertainties persist regarding long-term efficacy and the optimal technique. Ethanol sclerotherapy is best considered a complementary tool within individualized care, particularly for women prioritizing fertility preservation or those at risk from repeated surgeries. Future well-designed trials are needed to define its role more precisely and establish standardized protocols.
